# SpaGene: A Deep Adversarial Framework for Spatial Gene Imputation

**DOI:** 10.34133/csbj.0102

**Published:** 2026-05-15

**Authors:** Aishwarya Budhkar, Juhyung Ha, Qianqian Song, Jing Su, Xuhong Zhang

**Affiliations:** ^1^ Department of Computer Science, Indiana University Bloomington, Bloomington, IN, USA.; ^2^ Department of Health Outcomes and Biomedical Informatics, College of Medicine, University of Florida, Gainesville, FL, USA.; ^3^ Department of Biostatistics and Health Data Science, Indiana University School of Medicine, Indianapolis, IN, USA.

## Abstract

Integrating transcriptome-wide single-cell gene expression data with spatial context substantially enhances our understanding of tissue biology, cellular interactions, and disease progression. Although single-cell RNA sequencing (scRNA-seq) provides high-resolution gene expression data, it lacks crucial spatial context, whereas spatial transcriptomics techniques offer spatial resolution but are limited in the transcriptomics coverage. To address these limitations, integrating scRNA-seq and spatial transcriptomics data is essential. We introduce SpaGene, a deep learning framework designed to integrate scRNA-seq data and spatial transcriptomics data. SpaGene consists of 2 encoder–decoder pairs combined with 2 translators and 2 discriminators to effectively impute missing gene expression within spatial transcriptomics datasets. We benchmarked SpaGene against 6 representative methods across diverse datasets. Across dataset pairs under a controlled gene-holdout evaluation protocol, SpaGene improves average Pearson correlation coefficient and cell-wise structural similarity index and reduces root mean squared error compared to the evaluated baselines, indicating more accurate recovery of held-out spatial gene expression. Application of our model to lung tumor tissue revealed spatial patterns consistent with immune cell enrichment at tumor boundaries, restricted myeloid cell presence in adjacent normal regions, and improved detection sensitivity of microenvironment-driven pathways linked to immune neighborhoods. These findings provide insight into immune exclusion and tumor–immune interactions motivating further biological validation.

## Introduction

Recent advances in spatial transcriptomics (ST) techniques enable spatially resolved gene expression profiling at resolutions ranging from spots to single cells while retaining spatial information about cells. For example, using positional barcodes, detected RNA transcripts can be mapped back to their tissue region to retain spatial information [1]. Understanding transcriptional profiles at the cellular level aids in several ways, such as recognition of the similarities and differences within cell populations, which helps elucidate cellular heterogeneity, identification of cell development pathways, study of rare cell populations such as tumor cells, *etc.* [2,3]. The availability of commercial platforms such as the Vizgen MERSCOPE platform [4] and the NanoString CosMX Spatial Molecular Imager (SMI) platform [5] has enabled researchers and clinicians to access high-resolution ST data, facilitating the discovery of novel biological insights [3]. Spatial information, along with gene expression profiles, helps biologists understand complex cellular relationships and the resulting biological phenomenon [6]. For example, the NanoString platform enables spatial *in situ* detection of mRNA and proteins at the cellular and subcellular levels using formalin-fixed paraffin-embedded and fresh-frozen tissue samples [5]. Another imaging-based technique, MERSCOPE, uses multiplexed error robust fluorescence *in situ* hybridization (MERFISH) to capture the spatial distribution of RNA molecules at the single-cell level.

However, different ST techniques have their own limitations, such as less accurate capture of gene expression or limited spatial resolution. For example, imaging-based techniques such as MERFISH [7] and ouroboros single-molecule fluorescence in situ hybridization (osmFISH) [8] provide single-cell resolution with high accuracy. Still, they are limited to measuring gene expression for hundreds to a few thousand genes. Sequencing-based techniques such as Slide-seq [9] and 10x Visium [10] can detect thousands of genes but at lower spatial resolution than single-cell techniques and with lower capture efficiency. Similarly, the NanoString CosMX SMI platform [5] can profile thousands of genes, but the detection accuracy is low because of limitations of the technology.

Given that current ST technologies face notable limitations, there is a pressing need for computational strategies to enhance the quality and resolution of ST data. Prior to the development of the ST technology, single-cell RNA sequencing (scRNA-seq [SC]) emerged as a powerful tool for dissecting cellular heterogeneity and tracing cell lineages [2,3]. However, while SC offers detailed molecular profiles, it lacks spatial information, making it difficult to reconstruct the tissue architecture and cell–cell interactions within complex biological systems [1]. When combined with ST, SC serves as a valuable complementary modality, boosting the accuracy of spatially resolved transcriptomics analyses within individual tissue sections.

Several computational methods have been proposed to integrate SC with ST for gene expression imputation to overcome ST’s inherent limitations. For example, SpaGE (Spatial Gene Enhancement) [11] uses the PRECISE [12] domain adaptation algorithm to align datasets. After alignment, the *k*-nearest neighbor (*k*-NN) algorithm [13] is used to assign gene expression to spatially unmeasured locations using a weighted average of neighbors with positive cosine similarity. However, SpaGE faces limitations in handling complex datasets due to its reliance on *k*-NN and the PRECISE [12] algorithm, which uses linear dimensionality reduction. gimVI (Gene imputation with Variational Inference) [14] utilizes a deep generative model to integrate ST and SC datasets. The method learns a shared latent space of the input datasets and then uses posterior inference for gene imputation. gimVI can struggle to capture nuanced biological variations due to its limitations in latent space complexity and variability modeling. Tangram [15] integrates ST and SC data by learning a probabilistic assignment of cells to spatial spots and uses the mapping to impute gene expression at cellular resolution. However, its effectiveness depends on shared gene expression patterns to determine cell-to-spot mappings, which can limit performance in highly heterogeneous or novel biological contexts. VISTA [16] uses graph neural network model with variational modeling to predict unmeasured genes using SC data as reference. spRefine [17] uses a deep learning framework and genomic language models to denoise and impute ST data. spRefine does not explicitly use reference SC data for cross-domain alignment, and its performance can depend on the selected embedding model. stDiff [18] uses a diffusion model to understand gene expression relationships between SC and ST data and uses the learned relationships to predict unmeasured gene expression in ST data. In addition, stDiff focuses on denoising-based completion rather than bidirectional translation, which may limit flexibility under partial gene overlap and strong platform mismatch.

In this work, we introduce SpaGene, a translation-based deep learning method for predicting unmeasured genes in ST data by leveraging information from SC data. SpaGene utilizes an advanced encoder–decoder architecture supplemented by dedicated translator and discriminator modules to accurately impute missing gene expression within ST data. First, the encoder–decoder modules project each dataset into a low-dimensional latent space and reconstruct it back to the original dimension, learning to capture the distinctive characteristics of each dataset while preserving biologically relevant variation. Then, translators learn mappings between latent spaces, learning their shared features and their relationships. Discriminator modules further refine these mappings by encouraging the translated features to resemble the target data distribution. SpaGene is designed for ST–SC gene imputation where genes are unmeasured in ST but available in an SC reference. SpaGene performs translation in latent space to align domains under heterogeneous noise without forcing data-space correspondence on unshared genes. Translation is anchored by an identity constraint restricted to shared genes, and representation learning is decoupled from adversarial alignment by pretraining modality-specific autoencoders and freezing them during translator and discriminator training to improve stability and reduce oversmoothing. By leveraging the learned features from both datasets, SpaGene imputes unmeasured ST genes and expands transcriptome coverage, enabling more comprehensive downstream analysis such as spatial cellular interaction, pathway enrichment analysis, and microenvironment profiling. SpaGene’s contribution is the adaptation of cycle-consistent translation to ST–SC gene imputation under partial gene overlap and strong platform mismatch. In this setting, ST and SC share only a subset of genes, so SpaGene uses identity constraint restricted to shared genes to anchor translation. In addition, SpaGene decouples representation learning from adversarial alignment by pretraining modality-specific autoencoders and freezing them during translator training, which improves stability for the ST–SC imputation task.

## Results

### Overview of the SpaGene model

**Fig. 1.**
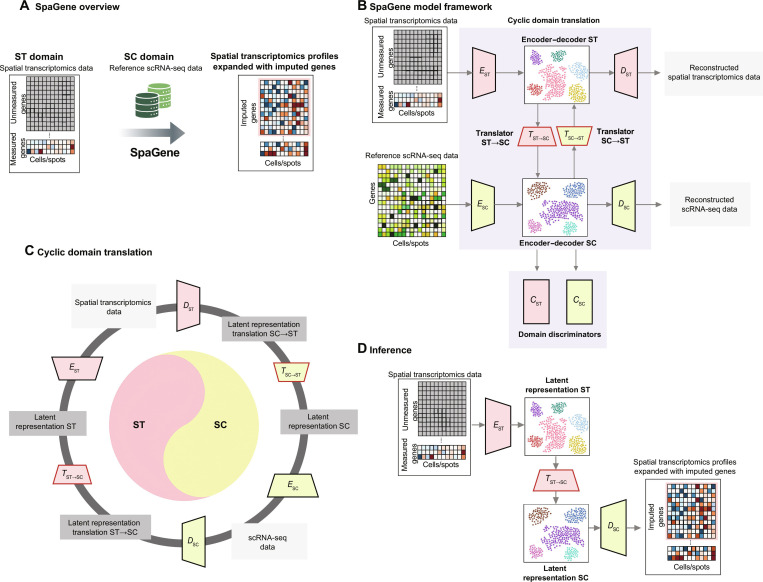
Overview of the SpaGene model. (A) SpaGene uses ST data and reference SC data as input to impute unmeasured genes in ST data. (B) SpaGene consists of 2 encoder–decoder pairs for ST and SC data each to learn the unique features of the datasets, 2 translators to map the latent representations between datasets, and 2 discriminators that guide the translators to generate realistic, biologically meaningful outputs. (C) Data are first embedded into a low-dimensional latent representation, followed by translation to the target dataset and back to the source to learn the shared features across datasets. (D) Missing gene expression in ST is imputed by translating the latent representation to the SC domain and decoding it to reconstruct the gene expression.

The primary function of the model is to enhance the limited ST data by expanding measured gene sets enriching the underlying biological information and, thus, facilitating improved downstream analysis and interpretation. Figure 1A illustrates the overall objective of SpaGene. SpaGene leverages reference SC data to predict unmeasured genes in ST datasets, resulting in spatial profiles enriched with imputed genes. As shown in Fig. 1B, the model comprises 3 components: encoder–decoder modules, translators, and discriminators. The model uses separate encoder–decoder networks for ST and SC datasets to capture and encode the unique dataset-specific features, mapping each dataset to compact, low-dimensional latent representations. This step is crucial to effectively capture essential characteristics of complex biological datasets and mitigate noise. The translators and discriminators then learn intricate nonlinear mappings between the latent spaces of ST and SC datasets. Translators are trained to convert representations from one dataset to another, while discriminators guide the translators to generate realistic outputs. This adversarial framework encourages translated outputs that are biologically plausible and closely resemble the target data distributions. The discriminators enforce stringent quality control on translated representations, helping translators to iteratively refine their outputs toward increased biological relevance. Figure 1C further elaborates on the domain translation process, where ST and SC data are projected into their latent representations and translated across domains. This bidirectional translation using the learned low-dimensional latent space representation helps the model learn shared features between the 2 datasets. As depicted in Fig. 1D during inference, ST data are projected into the learned latent space and translated into the SC domain. Finally, the gene expression is reconstructed, enhancing the ST data by expanding the measured gene expression. Thus, leveraging both shared and unique features of both datasets, SpaGene learns to translate ST data into the SC domain. ST and SC both measure gene expression but are produced by different technologies making their latent embeddings difficult to compare directly. To support the need for translator modules, we evaluated how well the local neighborhoods are preserved after translation in the learned latent spaces. As illustrated in Fig. S4, latent space translation from one domain to another yields relatively low neighborhood retentions, while cycle translation recovers a larger fraction of the original neighborhood. This supports the need for explicit translator modules between ST and SC latent spaces rather than using shared latent space.

### SpaGene consistently improves over existing methods across diverse datasets

To assess SpaGene’s ability to recover genes unmeasured in ST but available in SC reference, we benchmarked it against 6 representative methods—gimVI [14], SpaGE [11], Tangram [15], VISTA [16], spRefine [17], and stDiff [18]—across 8 diverse dataset pairs—MERFISH [7]_Moffitt [7], NanoString [5]_GSE [19], osmFISH [8]_AllenSSp [20], osmFISH_AllenVISp [21], osmFISH_Zeisel [22], seqFISH [23]_AllenVISp, STARmap [24]_AllenVISp, and Xenium_breast [25]_GSE_breast [25]—detailed in Materials and Methods section. Performance was evaluated using 5 metrics: Pearson correlation coefficient (PCC) [26], structural similarity index (SSIM) [26], root mean squared error (RMSE) [26], Wasserstein distance [27], and Jensen–Shannon (JS) divergence [26]. Across all 8 benchmarks, SpaGene showed the strong overall performance by capturing nonlinear cross-modal relationships. The performance results for PCC, RMSE, SSIM, Wasserstein distance, and JS metrics across the 8 dataset pairs are provided in Table S1. To evaluate the consistency of improvements across dataset pairs, we treated each dataset pair as the unit of replication (*n* = 8) and compared SpaGene to each baseline using paired analyses. For each competing method, its performance was compared to SpaGene on the same dataset pairs using paired *t* tests and Wilcoxon signed-rank tests to check whether SpaGene consistently improves over each baseline. SpaGene showed consistent improvements over SpaGE, gimVI, Tangram, VISTA, spRefine, and stDiff, and the corresponding false discovery rate (FDR)-corrected *P* values are reported in Table S2.

**Fig. 2.**
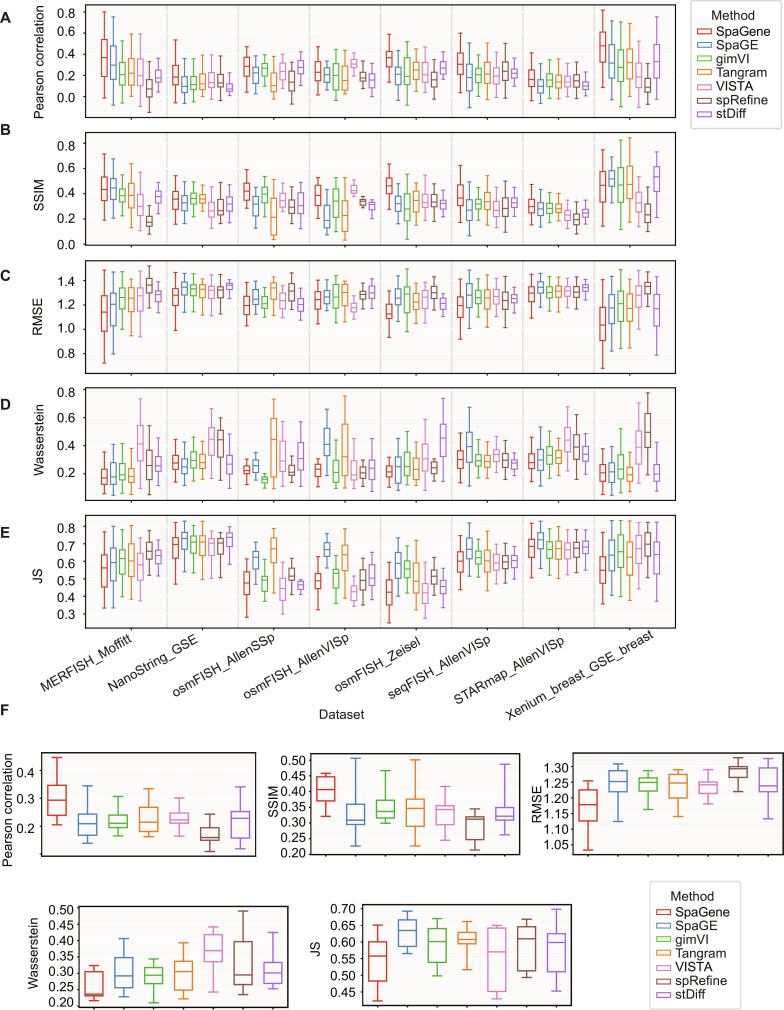
Performance evaluation across datasets. (A) Box plot of Pearson correlation coefficient (PCC) scores across 8 datasets for each method. (B) Box plot of structural similarity index (SSIM) scores across 8 datasets for each method. (C) Box plot of root mean squared error (RMSE) scores across 8 datasets for each method. (D) Box plot of Wasserstein scores across 8 datasets for each method. (E) Box plot of Jensen–Shannon (JS) scores across 8 datasets for each method. (F) Box plot of average PCC, SSIM, RMSE, Wasserstein, and JS scores for the 8 datasets for each method.

Figure 2A shows the PCC results for each method across the 8 datasets. SpaGene yields higher agreement with ground-truth spatial measurements under the same evaluation splits. For example, on the imaging-based MERFISH_Moffitt dataset pair, SpaGene achieved an average PCC of 0.381, compared with SpaGE (PCC = 0.345), gimVI (PCC = 0.242), Tangram (PCC = 0.273), VISTA (PCC = 0.244), spRefine (PCC = 0.119), and stDiff (PCC = 0.215). Similarly, on the NanoString_GSE dataset pair, SpaGene achieved an average PCC of 0.246, higher than SpaGE (PCC = 0.147), gimVI (PCC = 0.165), Tangram (PCC = 0.182), VISTA (PCC = 0.192), spRefine (PCC = 0.198), and stDiff (PCC = 0.119). Thus, SpaGene produces better alignment between predicted and measured expression profiles of individual genes, reflecting its capacity to learn complex, nonlinear mappings to reconstruct individual gene expression in spatial context. Figure 2B shows that SpaGene achieves strong SSIM scores across datasets. This demonstrates that it better preserves cell-wise expression structure across cells than competing methods. For example, for the osmFISH_AllenSSp dataset pair, SpaGene achieved an average SSIM of 0.414, better than SpaGE (average SSIM = 0.298), gimVI (average SSIM = 0.368), Tangram (average SSIM = 0.229), VISTA (average SSIM = 0.35), spRefine (average SSIM = 0.319), and stDiff (average SSIM = 0.313). Similarly, for the osmFISH_Zeisel dataset pair, SpaGene achieved an average SSIM of 0.447, higher than SpaGE (SSIM = 0.316), gimVI (SSIM = 0.299), Tangram (SSIM = 0.344), VISTA (average SSIM = 0.37), spRefine (average SSIM = 0.321), and stDiff (average SSIM = 0.323). These results show that SpaGene captures gene expression more accurately and also better preserves cell-wise patterns. Figure 2C presents RMSE values across all 8 datasets. SpaGene consistently reduces the average discrepancy between predicted and measured gene expression intensities across all datasets. For example, for the seqFISH_AllenVISp dataset pair, SpaGene achieved an average RMSE of 1.151, better than SpaGE (RMSE = 1.244), gimVI (RMSE = 1.257), Tangram (RMSE = 1.224), VISTA (RMSE = 1.246), spRefine (RMSE = 1.22), and stDiff (RMSE = 1.226). Similarly, for the STARmap_AllenVISp dataset pair, SpaGene achieved an average RMSE of 1.254, lower than SpaGE (average RMSE = 1.308), gimVI (average RMSE = 1.276), Tangram (average RMSE = 1.277), VISTA (average RMSE = 1.29), spRefine (average RMSE = 1.294), and stDiff (average RMSE = 1.315). Lower reconstruction error highlights SpaGene’s ability to more closely reproduce the observed relative expression structure across cells, minimizing systematic and random deviations. Figures 2D and E further shows that SpaGene remains competitive under the complementary-distribution-based metrics. Across all 8 datasets, SpaGene achieved the lowest average Wasserstein distance of 0.256 and the lowest average JS of 0.544, indicating improved agreement with measured expression distributions across cells (Fig. 2F).

Across all 8 datasets, SpaGene also achieved an average PCC of 0.303, 37.1% higher than SpaGE (PCC = 0.221), 37.7% higher than gimVI (PCC = 0.22), 32.9% higher than Tangram (PCC = 0.228), 32.9% higher than VISTA (PCC = 0.228), 79.3% higher than spRefine (PCC = 0.169), and 39% higher than stDiff (PCC = 0.218); and average SSIM of 0.403, 18.5% higher than SpaGE (SSIM = 0.34), 14.2% higher than gimVI (SSIM = 0.353), 19.2% higher than Tangram (SSIM = 0.338), 21.4% higher than VISTA (SSIM = 0.332), 39.9% higher than spRefine (SSIM = 0.288), and 20.3% higher than stDiff (SSIM = 0.335). SpaGene achieved a lower RMSE score of 1.167, 5.8% lower compared to SpaGE (RMSE = 1.239), 6% lower than gimVI (RMSE = 1.241), 5.4% lower than Tangram (RMSE = 1.234), 5.6% lower than VISTA (RMSE = 1.236), 9% lower than spRefine (RMSE = 1.283), and 6.5% lower than stDiff (RMSE = 1.248), as shown in Fig. 2F. Collectively, these results demonstrate SpaGene’s robust performance across diverse datasets including large-scale imaging-based datasets such as MERFISH profiling thousands of cells with large gene panels to targeted osmFISH assays where spatial measurements are limited to a small gene panel. Overall, the SpaGene framework shows consistent gains in PCC and SSIM and reduced RMSE under identical gene-holdout splits.

We additionally evaluated recovery of spatially variable genes (SVGs) using SPARK-X [28] to provide a quantitative analysis of spatial pattern preservation across methods. We used the significant SVGs identified by SPARK-X from the measured spatial data as the reference set and quantified the recovery from each method’s imputed data using area under the precision–recall curve (AUPRC). SpaGene showed competitive spatial-pattern recovery compared to other methods reported in Table S3.

We further examined the effect of shared gene overlap by retaining only 10%, 25%, 50%, 75%, or 100% of the shared genes for training on 2 representative dataset pairs, osmFISH_Zeisel and NanoString_GSE. In both cases, performance improved as more shared genes were retained (Fig. S1A). These results indicate that SpaGene performs best when more shared genes are available and the performance degrades gradually as overlap becomes sparser. We also examined the effect of ST data sparsity by masking 0%, 25%, 50%, 75%, or 90% of the originally nonzero expression values in the ST data for 2 representative dataset pairs, osmFISH_Zeisel and NanoString_GSE. In both cases, performance decreased as sparsity increased, with lower PCC and SSIM and higher RMSE under progressively sparser inputs (Fig. S1B). These results indicate that SpaGene maintains stable performance under moderate sparsity and degrades progressively as the ST data becomes sparser.

To assess whether SpaGene generated false-positive spatial patterns [29], we compared Moran’s *I* [30] between measured and imputed expression for each gene in the MERFISH_Moffitt dataset and summarized the spatial autocorrelation shift, ΔMoran’s *I*, defined as the difference between Moran’s *I* for the imputed gene expression and the measured gene expression. Across 141 held-out genes, the shift was generally modest (median ΔMoran’s *I* = 0.022, mean = 0.0145), and 78.7% genes remained within |ΔMoran’s *I*| ≤ 0.10, indicating that most genes remained close to measured spatial autocorrelation with mild overestimation for a subset of genes rather than strong false-positive spatial inflation across the full gene set (Fig. S2).

### SpaGene performance on the NanoString Lung9 rep1 dataset

**Fig. 3.**
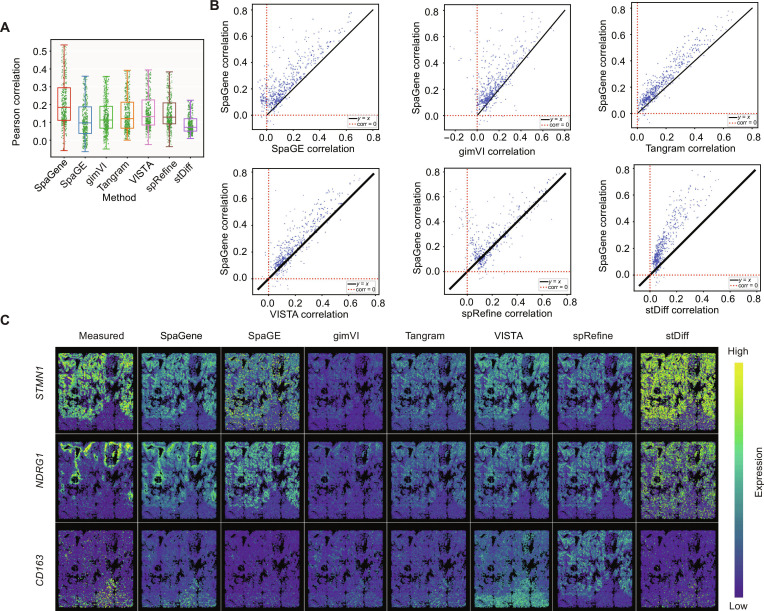
Performance evaluation on the NanoString Lung9 rep1 sample. (A) Box plot of Pearson correlation coefficient (PCC) for each competing method. (B) Scatter plots of PCC values for each imputed gene between SpaGene versus each competing method. (C) Spatial patterns of measured and imputed genes *STMN1*, *NDRG1*, and *CD163* for each method. The high/low color scale is provided as a visual guide and is not a shared quantitative scale across methods. In each panel, color intensity should be interpreted relative to the panel’s own expression range. Therefore, the visual comparison is intended to assess spatial pattern recovery rather than absolute expression magnitude across methods.

We applied SpaGene to integrate the NanoString Lung9 rep1 dataset with reference SC data to predict unmeasured spatial gene expression patterns, enriching the ST data. To rigorously evaluate the predictive performance of SpaGene, we conducted a 5-fold cross-validation, detailed in Materials and Methods section. In each fold, a subset of genes was held out, and the remaining genes were used to impute the spatial expression of the omitted genes. We compared our model’s performance with SpaGE, gimVI, Tangram, VISTA, spRefine, and stDiff using PCC between measured spatial gene expression and predicted values. Figure 3A shows the PCC results for the NanoString Lung9 rep1 tissue sample. SpaGene achieved an average PCC of 0.246, higher than SpaGE (average PCC = 0.147), gimVI (average PCC = 0.165), Tangram (average PCC = 0.182), VISTA (average PCC = 0.192), spRefine (average PCC = 0.198), and stDiff (average PCC = 0.119). We further conducted gene-level comparisons to provide detailed insights into the method performance. Figure 3B shows scatter plots comparing gene-wise PCC values between SpaGene and each competing method. We observed that most of the data points lie above the *y = x* line, demonstrating that SpaGene performs better than the competitors.

Furthermore, we visually assessed the spatial expression patterns of selected imputed genes with complex spatial patterns. Figure 3C illustrates measured and imputed spatial patterns of *STMN1*, *NDRG1*, and *CD163* genes in the NanoString Lung9 rep1 sample for SpaGene, SpaGE, gimVI, Tangram, VISTA, spRefine, and stDiff. Compared with the other methods, SpaGene produced more spatially coherent patterns that more closely matched the measured spatial organization. For example, SpaGene better recovered the spatial patterns for *NDRG1* and *CD163* than the compared methods: SpaGE showed a noisier pattern for *STMN1* and weaker recovery for *NDRG1* and *CD163*; gimVI produced smoother expression pattern for *STMN1*, and Tangram only broadly captured the spatial expression pattern for *NDRG1*; VISTA captured some broad spatial trends but showed less localized recovery, while spRefine and stDiff showed weaker spatial recovery overall, with stDiff appearing noisier. These examples illustrate that SpaGene can recover complex spatial expression patterns for representative genes under the same training and evaluation protocol.

### SpaGene performance on the STARmap dataset

**Fig. 4.**
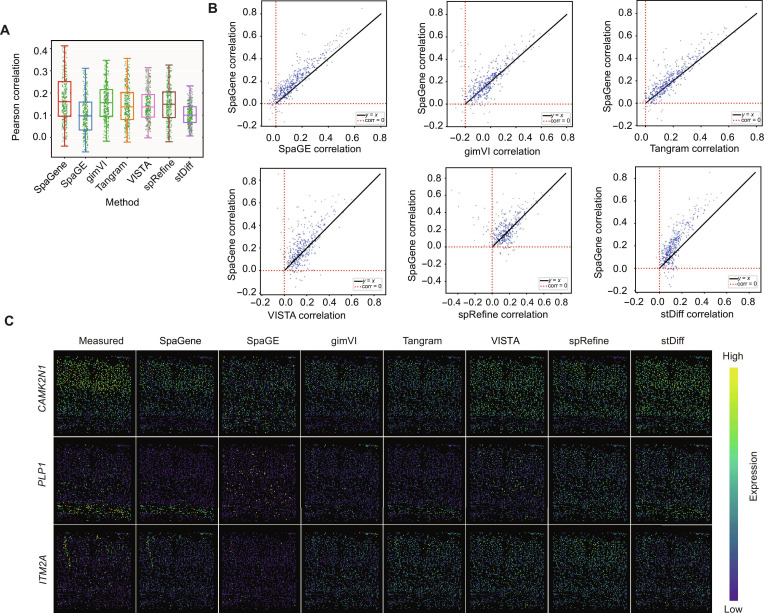
Performance evaluation on the STARmap data. (A) Box plot of Pearson correlation coefficient (PCC) is shown for each competing method. (B) Scatter plots of PCC values for each imputed gene between SpaGene versus each competing method. (C) Spatial patterns of measured and imputed genes *CAMK2N1*, *PLP1*, and *ITM2A* for each method. The high/low color scale is provided as a visual guide and is not a shared quantitative scale across methods. In each panel, color intensity should be interpreted relative to the panel’s own expression range. Therefore, the visual comparison is intended to assess spatial pattern recovery rather than absolute expression magnitude across methods.

Similarly, we evaluated the performance of SpaGene on the STARmap_AllenVISp dataset in predicting unmeasured spatial gene expression patterns. To assess the predictive accuracy, we used 5-fold cross-validation and quantified the performance using the PCC metric. Figure 4A summarizes the PCC results for the STARmap data, where SpaGene achieved an average PCC of 0.205, compared with SpaGE (PCC = 0.139), gimVI (PCC = 0.18), Tangram (PCC = 0.179), VISTA (average PCC = 0.164), spRefine (average PCC = 0.157), and stDiff (average PCC = 0.133). This improvement highlights the strong predictive power of SpaGene. To examine gene-level performance, Fig. 4B presents scatter plots comparing SpaGene’s PCC values with those of the other methods. Across all competitors, more data points lie above *y = x* line, which demonstrates that SpaGene often yields higher PCCs for a large proportion of genes.

In addition to quantitative performance, we evaluated the spatial fidelity of imputed gene expression patterns. Figure 4C displays spatial patterns of 3 representative imputed genes, *CAMK2N1*, *PLP1*, and *ITM2A*, by showing the ground truth STARmap measurements alongside predicted expression patterns for SpaGene, SpaGE, gimVI, Tangram, VISTA, spRefine, and stDiff. We observed that gene expression imputed by SpaGene more closely replicated the measured spatial distributions compared to the competitors. For example, for *CAMK2N1*, the broad expression pattern is captured by several methods, but SpaGene better preserved the spatial heterogeneity of the measured expression. For *PLP1*, SpaGene closely recovers the bottom-enriched spatial pattern, gimVI and Tangram capture the pattern reasonably well, whereas SpaGE, VISTA, spRefine, and stDiff showed weaker localization. For *ITM2A*, SpaGene showed a more consistent spatial pattern with measured expression, while several other methods produced less localized spatial pattern. Together, these visual and quantitative results indicate that SpaGene yields higher agreement with measured spatial patterns than the compared methods under the same evaluation protocol.

### SpaGene leverages imputed genes to uncover hidden biological insights

**Fig. 5.**
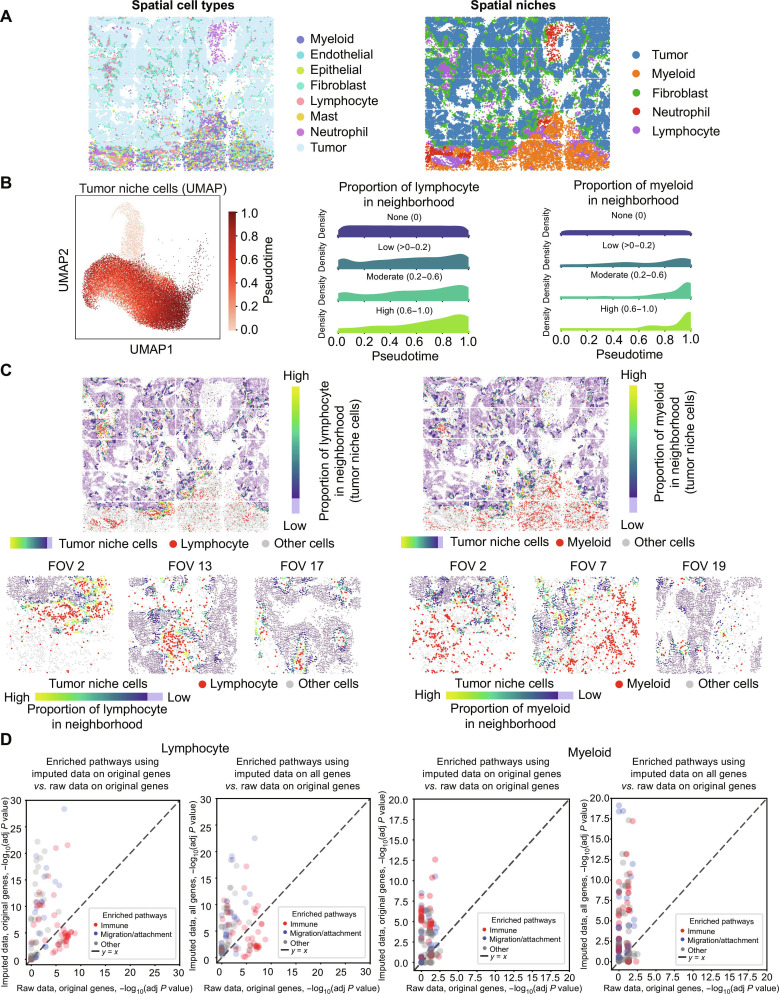
Downstream analysis using imputed data. (A) Spatial niche analysis. Spatial plot of cells colored by cell type, Spatial plot of cells colored by spatial niche: tumor, myeloid, fibroblast, neutrophil, and lymphocyte. (B) Pseudotime and immune neighbor association among tumor niche cells. Uniform Manifold Approximation and Projection (UMAP) for tumor niche cells colored by the inferred pseudotime, and ridge density plots showing distribution of tumor niche cells along pseudotime, grouped on the basis of their lymphocyte and myeloid cell neighbor proportion (none [0], low [>0 to 0.2], moderate [0.2 to 0.6], and high [0.6 to 1.0]). (C) Immune neighborhood for tumor niche cells. Spatial plot of cells colored by proportion of lymphocyte in neighborhood (left) and proportion of myeloid in neighborhood (right) for tumor niche cells with immune cells of interest highlighted in red and other cells in gray. Representative fields of view (FOVs) for lymphocyte (FOVs: 2, 13, and 17) and myeloid (FOVs: 2, 7, and 19) neighbor proportions for tumor niche cells colored from high (yellow) to low (purple), immune cells in red, and other cells in gray. (D) Pathway enrichment comparisons. Scatter plots of pathway enrichment significance (−log_10-_adjusted *P* value) detected using imputed versus raw expression data for lymphocyte (left 2 panels) and myeloid (right 2 panels): imputed original (only measured genes in raw data) versus raw (first column) and imputed all (both measured genes in raw data and newly imputed genes) versus raw (second column). Points in scatter plots denote pathways colored by annotation–immune (red), migration/attachment (blue), and other (gray), and the dashed line indicates the *y* = *x* line.

We investigated how the local immune microenvironment influences tumor cell states in the NanoString Lung9 rep1 sample using imputed gene expression profiles with spatial neighborhood-based analysis highlighting patterns for further validation. First, we defined spatial niches using Seurat v5 [31] based on each cell’s local neighborhood composition using *k*-NNs. Five major niches in the microenvironment were identified: tumor, myeloid, fibroblast, neutrophil, and lymphocyte. Figure 5A displays the spatial plot of cells with cell types and identified spatial niches that capture functionally distinct regions within the tissue.

To study tumor cell progression, we applied SpaTrack [32] to infer pseudotemporal trajectories within tumor niche cells. Figure 5B presents Uniform Manifold Approximation and Projection (UMAP) embeddings of tumor niche cells colored by inferred pseudotime and ridge density plots depicting distributions for these cells along pseudotime, grouped by their lymphocyte and myeloid cell neighbor proportion (none [0], low [>0 to 0.2], moderate [0.2 to 0.6], and high [0.6 to 1.0]). The ridge density plots reveal that tumor niche cells at later pseudotime are enriched in immune-rich neighborhoods. These patterns are consistent with an association between immune infiltration and tumor state transitions [33,34]. To spatially validate these patterns, we visualize the spatial plot of tumor niche cells in Fig. 5C. Tumor niche cells are colored by their lymphocyte and myeloid neighbor proportion, with immune cells of interest highlighted in red and all other cells shown in gray. Lymphocyte-rich neighborhoods are seen to be localized near tumor boundaries, while myeloid-rich neighborhoods are predominantly found in supporting tissue around the tumor. Representative fields of view (FOVs) illustrate immune enrichment at the tumor-normal interface and limited immune penetration into tumor regions, with myeloid cells accumulating in the normal tissue around the tumor.

Next, we investigate molecular pathways associated with immune infiltration. Local immune cell abundance can drive transcriptional changes in neighboring cells [35,36]. Therefore, we used the proportion of local immune neighbors for tumor niche cells to select genes for pathway analysis to capture both cell-specific and microenvironmentally regulated pathways that might not be captured by cell type information alone. Specifically, we computed PCC between gene expression and local immune neighbor proportions (lymphocyte and myeloid) for each tumor niche cell. Genes with moderate, biologically meaningful correlations were selected independently from 3 data subsets: raw gene expression, imputed gene expression with genes present in raw data, and all imputed gene expression. Using genes selected from each data subset, we performed pathway enrichment using Reactome and Gene Ontology gene sets from the MSigDB database collection [37,38]. We identified significant pathways, combined the top 20 enriched pathways for each data subset, and manually annotated them as immune, cell migration/attachment-related, or other. Figure 5D shows scatter plots comparing enrichment significance (−log_10_ adjusted *P* value) between raw and imputed data. We observed that using imputed expression increased the sensitivity for the detection of immune and cell migration/attachment-related pathways compared with raw expression. Moreover, inclusion of newly imputed genes increased the significance of detected pathways compared with using imputed expression only for genes already measured in raw data. Recent works have highlighted that downstream pathway analysis can benefit from improved enrichment strategies, suggesting that more accurate imputation and robust enrichment analysis are important for novel biological discoveries [39].

To further evaluate whether SpaGene preserves spatial structure for downstream analysis, we performed clustering using the Leiden algorithm [40] and SVG analyses using Moran’s *I* and SPARK-X on NanoString Lung9 rep1 sample. For clustering, we compared 3 data subsets: raw gene expression, imputed gene expression with genes presents in raw data, and all imputed gene expression. We evaluated the clustering performance using adjusted rand index (ARI), normalized mutual information (NMI), and silhouette score. The performance was preserved when using imputed gene expression with genes present in raw data measured by (ARI: 0.349, NMI: 0.492, silhouette score: 0.064) relative to the raw gene expression (ARI: 0.343, NMI: 0.451, silhouette score: 0.052), while all imputed gene expression further improved separation of cell clusters (ARI: 0.460, NMI: 0.487, silhouette score: 0.148) (Fig. S3A). As an additional comparison across methods, we performed clustering using the imputed gene expression from each method and evaluated agreement with the ground truth cell type labels using ARI. SpaGene achieved the highest ARI (0.46), compared with SpaGE (0.207), gimVI (0.34), Tangram (0.424), VISTA (0.406), spRefine (0.42), and stDiff (0.158). For SVG evaluation, we compared raw gene expression and imputed gene expression for genes present in raw data using Moran’s *I* and SPARK-X. Moran’s *I* values were highly concordant between raw and imputed data, indicating a strong preservation of spatial autocorrelation. SPARK-X also identified substantially overlapping 834 significant SVGs for raw data and 877 for imputed data at an FDR of <0.05 and a Jaccard overlap of 0.746 (Fig. S3B). Together, these analyses show that SpaGene preserves the original spatial structure in measured gene expression data, while the imputed data provide additional signal that can improve downstream clustering.

## Discussion

Spatial single-cell transcriptomics data play an important role in understanding complex tissue structures and functions by revealing spatially resolved gene expression at single-cell resolution. However, only a limited subset of genes is typically captured, constraining comprehensive biological interpretation. Accurate gene imputation using single-cell reference data can address this limitation and aid in advancing spatial biology, enabling insights into cellular heterogeneity, interactions, and underlying tissue mechanisms. Beyond gene imputation to enhance data for downstream analysis, recent computational works in ST also focus on spatial domain identification and SVG detection highlighting the growing need for accurate and biologically relevant analysis tools for spatial data [41,42].

The primary objective of SpaGene is to enhance the ST data by expanding gene coverage beyond the assayed set through effective integration with reference SC data facilitating improved downstream analysis. The framework consists of 2 encoder–decoder networks, 2 translators, and 2 discriminators. Separate encoders learn robust latent space representations from ST and SC data. Two translators are trained to translate data between domains. To enhance ST data, its latent representation is translated into the SC domain by a dedicated translator module. The translated representation is then used to reconstruct comprehensive gene expression profiles using the SC decoder module. By learning nonlinear cross-domain relationships, SpaGene produces imputations that preserve expression patterns. Thus, SpaGene serves as an advanced computational approach by expanding ST gene coverage supporting downstream spatial analyses.

Although both SC and ST measure gene expression, they use fundamentally different experimental processes. SC is characterized by dissociation-induced dropout, amplification noise, and library-size variation [43], while ST technologies are influenced by optical background, platform-dependent biases, and segmentation errors [44]. Forcing both modalities into a shared latent space assumes that these distortions are comparable, which can lead to oversmoothing and reduced accuracy in prediction of unmeasured genes. Thus, a key design choice is to use separate latent embeddings to allow each modality to capture its own technology-specific expression patterns, while translators enforce biological consistency across domains. This is supported by Fig. S4 where translation between ST and SC domains changes nearest-neighbor relationships, while cycle translation recovers local neighborhoods, indicating that mapping between the domains is nonidentity and explicit translation is required between distinct latent spaces. This design separates biological signal from platform-specific noise and supports cross-modal gene imputation. We chose to freeze the pretrained autoencoders during translator training to stabilize modality-specific latent representations before adversarial alignment since jointly updating representation learning and adversarial translation can cause the latent spaces to drift during training. The 2-stage training strategy showed better held-out performance on representative dataset pairs as shown in Table S4 and validation correlation trajectories are shown in Fig. S5. The observed variation in performance across dataset pairs reflects fundamental differences in both measurement technology and available information between the ST and SC domains. In particular, dataset difficulty is determined by number of shared genes available for anchoring, the degree of technology-specific noise and sparsity such as imaging-based assays versus droplet-based SC, and biological heterogeneity of the tissue [44,45]. Consequently, datasets with limited gene panels and strong platform mismatch, such as osmFISH and NanoString, pose more challenging translation problems than high-plex or region-matched pairs such as MERFISH_Moffitt and seqFISH_AllenVISp. Under these conditions, SpaGene shows more consistent performance than methods based on shared-gene alignment or a single latent space, highlighting the benefit of explicit cross-modal translation.

Biologically, SpaGene provides a more comprehensive view of the tumor immune microenvironment using the expanded spatial transcriptomics profile, supporting analysis of cellular interactions and biological pathways that may be underrepresented when using measured genes alone. In the NanoString Lung9 rep1 sample, the enriched transcriptome enabled detection of spatial patterns such as lymphocytes clustering at tumor boundaries and reduced myeloid infiltration at the boundary between tumor and normal region. The expanded gene coverage highlighted associations between the immune neighborhood and tumor cell state transitions, offering insights into how immune pressure influences tumor evolution. Moreover, the model improves detection of microenvironment-driven pathways, particularly immune and cell migration/attachment pathways, relative to the raw data. These findings indicate that SpaGene utilizes the expanded transcriptome to find patterns of tumor evolution and to improve the detection of biologically relevant pathways.

While SpaGene has demonstrated improved performance in integrating ST and SC datasets, it has several limitations and holds potential for future development. SpaGene currently relies solely on transcriptomics information and does not incorporate complementary modalities such as tissue morphology and protein abundance, which can provide additional context about cell state and tissue organization. Although SpaGene’s cross-modal translation framework is designed to align datasets with different measurement characteristics, extremely sparse, noisy datasets, or biologically mismatched datasets may still impose limits on the amount of recoverable biological signal. SpaGene could be further improved through adaptive training strategies based on data quality. Another limitation is that SpaGene does not currently provide imputation confidence estimates for truly unmeasured genes. Although held-out gene analysis can be used to evaluate reconstruction reliability and possible spatial overestimation when ground truth is available, it does not directly provide calibrated confidence for genes that are unmeasured in ST. False-positive spatial patterns can be addressed by evaluating spatial autocorrelation shifts on held-out genes where measured ground truth is available and by interpreting genes with larger spatial autocorrelation more cautiously in downstream analysis. In the future, post hoc uncertainty estimation using frameworks such as TISSUE [46] could be incorporated to support uncertainty-aware downstream use of imputed expression. A limitation of the current study is that the biological validation remains computational. Although the tumor microenvironment analysis revealed spatially coherent immune-neighborhood patterns and biological pathways, which are supported with known literature, these findings should be interpreted as hypothesis generating rather than definitive experimental confirmation. Orthogonal validation using approaches such as protein staining would further strengthen the biological conclusions and will be an important direction for future work. Integration of additional data modalities such as imaging, proteomics, epigenomics, or metabolomics would further constrain predictions and enable a comprehensive understanding of tissue morphology and underlying biological processes. Another important avenue for future development is enhancing model interpretability through advanced explainability techniques [47], such as attention frameworks [48], integrated gradients [49], and layer-wise relevance propagation [50], which could increase trust in model outputs. Greater transparency into how predictions are made may facilitate the discovery of novel biomarkers, provide deeper insights into cellular mechanisms, and support more effective translation of computational findings into biological knowledge [47]. Overall, SpaGene is an advanced tool for ST, improving gene imputation and integration capabilities to drive forward research in spatial biology.

## Materials and Methods

### Data processing

The following ST and SC dataset pairs are used for performance evaluation: MERFISH_Moffitt, NanoString_GSE, osmFISH_AllenSSp, osmFISH_AllenVISp, osmFISH_Zeisel, seqFISH_AllenVISp, STARmap_AllenVISp, and Xenium_breast_GSE_breast. A comprehensive summary of dataset statistics is provided in Table S5. For both ST and SC datasets, genes with detection rate less than 0.05 were filtered where detection rate is the fraction of cells with nonzero expression for a gene. For both datasets, cells with a detection rate of <0.1 were removed to reduce noise and computational load where detection rate is the fraction of genes with nonzero expression within a cell. Next, 2,000 highly variable genes were selected for SC using Scanpy [51]. To reduce the influence of extreme values, outliers exceeding 2 standard deviations above the mean for each feature were clipped to the mean plus 2 standard deviations. This allowed to retain potentially informative samples while limiting the effect of extreme feature values. Finally, we applied square root normalization by computing the square root of each expression value in the data to reduce data skewness. We used square root normalization as a mild variance stabilizing transformation for continuous representation learning, which reduces skewness while preserving dynamic range more gently than log transformation.

### The SpaGene model

SpaGene consists of 2 domain-specific encoder–decoder autoencoders, 2 latent-space translators, and 2 latent-space discriminators. Each encoder–decoder pair follows the AutoEncoder [52] framework, which uses an encoder function to reduce the original data features into a low-dimensional feature space and a decoder function to reconstruct the data from that low-dimensional feature space. The encoder is a multilayer fully connected neural network with nonlinear activations that compresses the given gene expression into a latent space representation. The decoder is a neural network that reconstructs the original gene expression from the latent space representation through successive nonlinear transformations. Translators facilitate domain adaptation by translating the latent space representation between the ST and SC domains. Discriminators are used for distinguishing whether a latent space representation originates from the source domain or was generated by a translator. The trained discriminator provides adversarial loss [53] that guides the translators to generate more realistic outputs.

### Encoder–decoder

SpaGene consists of 2 encoder–decoder modules, one for the ST and one for the SC domain. Each module comprises an encoder E that maps an input expression vector x to a low-dimensional latent representation z and decoder D that maps z back to a reconstruction $\hat{x}$. For the ST encoder, a fully connected multilayer perceptron (MLP) with hidden layers of size 2,048, 1,024, and 512 maps input gene expression to a latent dimension of 256. The ST decoder uses an MLP with hidden layers of size 512, 1,024, and 2,048 to reconstruct the input. For the SC encoder, the MLP uses hidden layers of size 2,048, 1,024, and 512 to map the input gene expression to a latent dimension of 512, and the SC decoder uses hidden layers of size 512, 1,024, and 2,048 to reconstruct the input. When applied to the ST and SC domains, the encoders produce $z_{\mathrm{ST}}=E_{\mathrm{ST}}\left(x_{\mathrm{ST}}\right)$ and $z_{\mathrm{SC}}=E_{\mathrm{SC}}\left(x_{\mathrm{SC}}\right)$, and the decoders reconstruct ${\hat{x}}_{\mathrm{ST}}=D_{\mathrm{ST}}\left(z_{\mathrm{ST}}\right)$ and ${\hat{x}}_{\mathrm{SC}}=D_{\mathrm{SC}}\left(z_{\mathrm{SC}}\right)$. To train the encoder–decoder, reconstruction error is minimized as follows:

minE,DExMSEDEx,x (1)

where $\mathrm{MSE}\left(x,\;y\right)$ stands for the mean-squared-error loss.

Both domain-specific modules are trained independently to minimize reconstruction error. Once the encoder–decoders are trained, their weights are frozen.

### Translator

The model comprises 2 directional translators: $T_{\mathrm{ST}\to \mathrm{SC}}$ that maps ST latent space $z_{\mathrm{ST}}$ to SC latent space $z_{\mathrm{ST}\to \mathrm{SC}}$ and $T_{\mathrm{SC}\to \mathrm{ST}}$ that maps SC latent space $z_{\mathrm{SC}}$ to ST latent space $z_{\mathrm{SC}\to \mathrm{ST}}$. The $T_{\mathrm{ST}\to \mathrm{SC}}$ translator maps a 256-dimensional latent vector to a 512-dimensional latent vector using hidden layers of size 512, 512, 512, 1,024, and 1,024. The $T_{\mathrm{SC}\to \mathrm{ST}}$ translator maps a 512-dimensional latent vector to a 256-dimensional latent vector using hidden layers of size 512, 256, 256, 128, and 128. Here,

$$z_{\mathrm{ST}\to \mathrm{SC}}=T_{\mathrm{ST}\to \mathrm{SC}}\left(z_{\mathrm{ST}}\right),\;z_{\mathrm{SC}\to \mathrm{ST}}=T_{\mathrm{SC}\to \mathrm{ST}}\left(z_{\mathrm{SC}}\right)$$ (2)

The objective of these translators is to generate realistic latent representations in the translated domains.

To assess how translation affects local structure, we measure neighborhood preservation in latent space using cosine distance. For each cell, we define its source neighborhood as *k* = 15 nearest neighbors in the original latent space. After translation (ST → SC or SC→ ST) or cycle translation (ST → SC → ST or SC→ ST → SC), we compute neighborhood recall (*k* = 15, *m* = 45) as the fraction of those 15 source neighbors that remain among the *m* = 45 nearest neighbors of the translated or cycled embedding. We report the mean neighborhood recall across cells over training epochs with separate curves for each fold. We use a top 45 recovery window to allow small neighbor rank changes after translation while still evaluating local neighborhood preservation.

### Discriminator

We use 2 discriminators: $C_{\mathrm{ST}}$ is trained to give higher scores to latent representation generated from real ST data and lower scores to embeddings generated using the translator $T_{\mathrm{SC}\to \mathrm{ST}}$. Similarly, $C_{\mathrm{SC}}$ is trained to distinguish the real SC embeddings from those produced by the translator $T_{\mathrm{ST}\to \mathrm{SC}}$. Discriminators provide feedback that guides translators to generate more realistic representations through adversarial learning. $C_{\mathrm{ST}}$ and $C_{\mathrm{SC}}$ use MLP with hidden layers of size 256, 128, 128, and 64.

### Loss function

SpaGene combines multiple objectives, including cycle consistency, identity preservation, and adversarial domain alignment. The loss design follows general structure of cycle-consistent adversarial translation. Three loss functions are used, including cycle loss [54], identity loss (ID loss) [55], and generative adversarial network (GAN) loss [55].

Cycle loss ensures that translating the latent space to the other domain and back to the original domain preserves the original gene expression. The cycle-reconstructed expression is defined as:

$$\begin{array}{c} x_{\mathrm{ST}}^{\mathrm{cyc}}=D_{\mathrm{ST}}\left(T_{\mathrm{SC}\to \mathrm{ST}}\left(T_{\mathrm{ST}\to \mathrm{SC}}\left(z_{\mathrm{ST}}\right)\right)\right),\;\\ x_{\mathrm{SC}}^{\mathrm{cyc}}=D_{\mathrm{SC}}\left(T_{\mathrm{ST}\to \mathrm{SC}}\left(T_{\mathrm{SC}\to \mathrm{ST}}\left(z_{\mathrm{SC}}\right)\right)\right) \end{array}$$ (3)

and the cycle loss is defined as:

$$L_{\mathrm{cyc}}=\mathrm{MSE}\left(x_{\mathrm{ST}}^{\mathrm{cyc}},\;x_{\mathrm{ST}}\right)+\mathrm{MSE}\left(x_{\mathrm{SC}}^{\mathrm{cyc}},x_{\mathrm{SC}}\right)$$ (4)

where $x_{\mathrm{ST}}$ and $x_{\mathrm{SC}}$ denote the original gene expression vectors in the ST and SC domains, $z_{\mathrm{ST}}=E_{\mathrm{ST}}\left(x_{\mathrm{ST}}\right)$ and $z_{\mathrm{SC}}=E_{\mathrm{SC}}\left(x_{\mathrm{SC}}\right)$ denote the latent embeddings produced by ST and SC encoders, $T_{\mathrm{ST}\to \mathrm{SC}}$ and $T_{\mathrm{SC}\to \mathrm{ST}}$ are translators between the 2 latent spaces, $D_{\mathrm{ST}}$ and $D_{\mathrm{SC}}$ are ST and SC decoders, $x_{\mathrm{ST}}^{\mathrm{cyc}}$ and $x_{\mathrm{SC}}^{\mathrm{cyc}}$ are the cycle-reconstructed gene expression vectors, $\mathrm{MSE}\left(x,y\right)$ is the mean-squared-error loss function.

ID loss uses the Pearson correlation between the original gene expression and the translated gene expression. Since the dimensions of the translated gene expression and original gene expression are different, backpropagation for ID loss is restricted to the shared gene indices that exist in both domains, so that only shared genes constrain translation while unshared genes can be inferred. By minimizing this loss, the translator learns to generate gene expression that align closely with the target domain values. Thus, in our model, the ID loss is defined as:

$$L_{\mathrm{ID}}=\left(1-\rho \left(D_{\mathrm{SC}}\left(z_{\mathrm{ST}\to \mathrm{SC}}\right),\;x_{\mathrm{ST}}\right)\right)+\left(1-\rho \left(D_{\mathrm{ST}}\left(z_{\mathrm{SC}\to \mathrm{ST}}\right)\;,x_{\mathrm{SC}}\right)\right)$$ (5)

where $x_{\mathrm{ST}}$ and $x_{\mathrm{SC}}$ denote the original gene expression vectors in the ST and SC domains, $z_{\mathrm{ST}\to \mathrm{SC}}$ = $T_{\mathrm{ST}\to \mathrm{SC}}\left(z_{\mathrm{ST}}\right)$ and $z_{\mathrm{SC}\to \mathrm{ST}}$ = $T_{\mathrm{SC}\to \mathrm{ST}}\left(z_{\mathrm{SC}}\right)$ denote translated latent embeddings, and ρ is the Pearson correlation computed over the shared gene set.

GAN loss introduces an adversarial objective where domain-specific discriminators operate on latent embeddings and are trained to assign higher scores to the latent space representations that originate from a domain and lower scores to translated embeddings. Each translator maps latent representations from one domain to the other. Translators are trained to increase discriminator scores for the translated embeddings, encouraging translated latent embeddings to match the target latent distribution.

The translator is trained to fool the discriminator by generating representations $x_{\text{fake}}\;$that are indistinguishable from latent representations originating from the source domain $x_{\text{real}}$ with the loss defined as:

$$L_{\mathrm{GAN}}=-E_{x_{\text{fake}}}C\left(x_{\text{fake}}\right)$$ (6)

Specifically, for 2 translators the GAN loss is given as:

$$L_{\mathrm{GAN}}=-E_{z_{\mathrm{SC}\to \mathrm{ST}}}\left[C_{\mathrm{ST}}\left(T_{\mathrm{SC}\to \mathrm{ST}}\left(z_{\mathrm{SC}}\right)\right)\right]-E_{z_{\mathrm{ST}\to \mathrm{SC}}}C_{\mathrm{SC}}\left[\left(T_{\mathrm{ST}\to \mathrm{SC}}\left(z_{\mathrm{ST}}\right)\right)\right]$$ (7)

where $C_{\mathrm{ST}}$ and $C_{\mathrm{SC}}$ denote the discriminators for the ST and SC latent spaces, E denotes the expectation over samples from the indicated latent distribution, and $T_{\mathrm{SC}\to \mathrm{ST}}\left(z_{\mathrm{SC}}\right)$ and $T_{\mathrm{ST}\to \mathrm{SC}}\left(z_{\mathrm{ST}}\right)$ are translated latent embeddings.

The discriminators are trained using hinge loss, enforcing a margin separator to distinguish between representations from the source domain and those generated by a translator as follows:

$$L_{\text{disc}}=E_{x_{\text{real}}}\left[\max\left(0,1-C\left(x_{\text{real}}\right)\right)\right]+E_{x_{\text{fake}}}\left[\max\left(0,1+C\left(x_{\text{fake}}\right)\right)\right]$$ (8)

Thus, for our model, the discriminator loss is defined as:

$$\begin{array}{c} L_{\text{disc}}=E_{z_{\mathrm{ST}}}\left[\max\left(0,1-C_{\mathrm{ST}}\left(z_{\mathrm{ST}}\right)\right)\right]+E_{z_{\mathrm{SC}}}\left[\max\left(0,1+C_{\mathrm{ST}}\left(T_{\mathrm{SC}\to \mathrm{ST}}\left(z_{\mathrm{SC}}\right)\right)\right)\right]\\ +E_{z_{\mathrm{SC}}}\left[\max\left(0,1-C_{\mathrm{SC}}\left(z_{\mathrm{SC}}\right)\right)\right]+E_{z_{\mathrm{ST}}}\left[\max\left(0,1+C_{\mathrm{SC}}\left(T_{\mathrm{ST}\to \mathrm{SC}}\left(z_{\mathrm{ST}}\right)\right)\right)\right] \end{array}$$ (9)

where $C_{\mathrm{ST}}$ and $C_{\mathrm{SC}}$ denote the discriminators for the ST and SC latent spaces, $z_{\mathrm{ST}}$ and $z_{\mathrm{SC}}$ are real latent embeddings from the ST and SC domains, $T_{\mathrm{SC}\to \mathrm{ST}}\left(z_{\mathrm{SC}}\right)$ and $T_{\mathrm{ST}\to \mathrm{SC}}\left(z_{\mathrm{ST}}\right)$ are translated embeddings, and E denotes the expectation over samples from the indicated latent distribution.

The translator loss combines ID loss, cycle loss, and GAN loss as follows:

$$L_{\text{trans}}=\lambda_{\mathrm{ID}}L_{\mathrm{ID}}+\lambda_{\mathrm{cyc}}L_{\mathrm{cyc}}+\lambda_{\mathrm{GAN}}L_{\mathrm{GAN}}$$ (10)

where $\lambda_{\mathrm{ID}}=1$, $\lambda_{\mathrm{cyc}}=1$, and $\lambda_{\mathrm{GAN}}=0.1$.

### Model inference

Gene expression from the ST domain $x_{\mathrm{ST}}$ is passed through the trained encoder $E_{\mathrm{ST}}$ to generate the latent space representation $z_{\mathrm{ST}}$.$z_{\mathrm{ST}}$ is then translated into the SC domain $z_{\mathrm{ST}\to \mathrm{SC}}$ using the trained translator $T_{\mathrm{ST}\to \mathrm{SC}}$. Finally, the translated latent space is passed through the trained decoder of the SC domain $D_{\mathrm{SC}}$ to generate the translated gene expression from the ST to SC domain ${\hat{x}}_{\mathrm{SC}}=D_{\mathrm{SC}}\left(z_{\mathrm{ST}\to \mathrm{SC}}\right)$.

### Experimental setup

The training is divided into 2 stages: In the first stage, the encoder–decoder networks are trained to efficiently encode and reconstruct gene expression, ensuring faithful compression and reconstruction of input data. Encoder–decoder pairs are trained independently using MSE reconstruction loss for 100 epochs. In the second stage, encoder–decoder weights are frozen, and translators and discriminators are trained to translate the latent representations between the ST and SC domains for 50 epochs. All networks are optimized using Adam optimizer with a batch size of 512. During autoencoder pretraining, encoder–decoders are optimized with a learning rate of 1 × 10^−3^. During translator training, translators are updated with a learning rate of 1 × 10^−3^, while discriminators are trained using a smaller learning rate of 5 × 10^−4^ to prevent adversarial instability and discriminator overpowering. All networks are implemented as MLPs composed of linear layers, followed by batch normalization, LeakyReLU [56] activation, and dropout (*P* = 0.2), which stabilizes optimization, prevents vanishing gradients, and reduces overfitting across all components of the model.

To ensure consistent evaluation, we used a single model architecture and loss configuration across all dataset pairs to avoid data-specific adjustment. We assessed the robustness of the fixed configuration using sensitivity analysis. Sensitivity results are provided in Tables S6 to S9. In cross-technology gene imputation, shared genes provide the only direct molecular correspondence between ST and SC domains. ID loss ensures that these genes remain stable during adversarial translation, preventing biologically inconsistent drift of shared gene expression while unmeasured genes are inferred. Consistent with this role, removing the ID loss caused the largest performance degradation. As shown in the loss ablation results in Tables S8 and S9, removing the ID loss caused the largest performance drop while removing the cycle loss also reduced performance but less severely, supporting the inclusion of both in the final objective. The network widths and latent dimensions were selected empirically using sensitivity analysis provided in Tables S6 and S7. In particular, the asymmetric latent dimensions of 256 for ST and 512 for SC provided a strong tradeoff between predictive performance and model complexity. Adversarial loss weight is set to 0.1 while keeping cycle and ID loss weights as 1 because the adversarial term is intended to provide a mild distribution matching signal and larger adversarial weights did not produce consistent gains and could reduce stability, whereas the cycle and identity terms more directly preserve cross-domain consistency and shared gene anchoring (Tables S6 to S6).

We evaluate gene imputation using 5-fold cross-validation defined on the set of genes shared between the ST and SC domains. For each dataset pair, $G_{\text{shared}}$ denotes shared genes between the ST and SC domains (MERFISH_Moffitt dataset pair: 141, NanoString_GSE dataset pair: 488, osmFISH_AllenSSp dataset pair: 26, osmFISH_AllenVISp dataset pair: 26, osmFISH_Zeisel dataset pair: 32, seqFISH_AllenVISp dataset pair: 411, STARmap_AllenVISp dataset pair: 242, Xenium_breast_GSE_breast dataset pair: 199). We partition $G_{\text{shared}}$ into 5 disjoint folds. In fold k, we designate onefold as held-out genes $G_{\text{test}}^{\left(k\right)}$, which are treated as unmeasured genes in ST and are removed, while the remaining genes $G_{\text{train}}^{\left(k\right)}$ in ST data were used as observed ST input genes for training. All ST gene-level loss terms are computed only on $G_{\text{train}}^{\left(k\right)}$, while predictions for $G_{\text{test}}^{\left(k\right)}$ are generated after training is complete for that fold. All model training, including autoencoder pretraining, translator, and discriminator training, is performed within each fold using only $G_{\text{train}}^{\left(k\right)}$ for ST supervision, and held-out genes are used only to compute evaluation metrics. Model performance is evaluated by comparing the measured and predicted gene expression profiles on the held-out genes. The performance is evaluated exclusively on held-out gene folds, providing an explicit control against overfitting to the measured gene set. The held-out genes are removed from the ST inputs and excluded from all ST-supervised loss terms. Held-out genes are only used for final evaluation against measured ST values. This protocol mirrors the practical scenario where a gene is unavailable in ST data but present in SC reference. We focus on gene imputation under partial gene overlap and platform mismatch where ST lacks a subset of genes that are present in an SC reference. We therefore compare against 6 representative methods SpaGE, gimVI, Tangram, VISTA, spRefine, and stDiff that can be evaluated under identical gene holdout splits without requiring cell type labels or additional modalities. The same gene-fold partitions were reused across all methods for comparison. For reproducibility, the source repositories and exact versions or commit used for all baseline methods are listed in Table S10.

The assumption underlying this benchmark is that once a shared gene is removed from the ST input, it serves as a proxy for an unshared gene because it must be predicted from the remaining shared genes and the SC reference. This proxy enables quantitative evaluation using the ST ground truth, but it does not guarantee identical recovery for all truly unshared genes. To assess robustness to sparse shared-gene overlap, we performed an additional reduced shared gene overlap analysis on representative dataset pairs. Starting from the original set of genes shared between ST and SC datasets, we retained only 10%, 25%, 50%, 75%, or 100% of the shared genes available during training and repeated the same cross-validation fold-based evaluation on the retained set. To assess robustness to increased ST data sparsity, we performed sparsity ablation analysis on representative dataset pairs. We randomly masked 0%, 25%, 50%, 75%, or 90% of the original nonzero entries to generate progressively sparser data and repeated the same cross-validation fold-based evaluation. Performance was quantified using PCC, SSIM, and RMSE.

### Evaluation metrics

Given N cells and G genes in the ST dataset, the measured expression for gene g as $x_{g}$, and the predicted expression for gene g as ${\hat{x}}_{g}$, we used the following metrics to assess the performance of our method.

#### Pearson correlation coefficient

The PCC [26] between each gene g in predicted expression by each method and the ground truth expression of ST is computed as follows:

$$\mathrm{PCC}\left(g\right)=\frac{\mathrm{Cov}\left(x_{g},\;{\hat{x}}_{g}\right)}{\sigma_{x_{g}}\sigma_{{\hat{x}}_{g}}}$$ (11)

where $\begin{array}{c} \begin{array}{c} \mathrm{Cov}\left(x_{g},{\hat{x}}_{g}\right)=\frac{1}{N}\sum_{i=1}^{N}\left(x_{g,i}-\mu_{g}\right)\left({\hat{x}}_{g,i}-{\hat{\mu }}_{g}\right),\mu_{g}=\frac{1}{N}\sum_{i=1}^{N} \end{array} \end{array}$ $x_{g,\;i},{\hat{\mu }}_{g}=\frac{1}{N}\sum_{i=1}^{N}{\hat{x}}_{g,\;i}$, and $\sigma_{x_{g}}\;\text{and}\;\sigma_{{\hat{x}}_{g}}\;$ are their standard deviations.

#### Structural similarity index

The SSIM [26] measures the similarity between measured and predicted gene expression. SSIM is computed after normalization of each gene across cells as follows:

$$x_{g}^{\prime }=\frac{x_{g}-\min\left(x_{g}\right)}{\max\left(x_{g}\right)-\min\left(x_{g}\right)},\;{\hat{x}}_{g}^{\prime }=\frac{{\hat{x}}_{g}-\min\left({\hat{x}}_{g}\right)}{\max\left({\hat{x}}_{g}\right)-\min\left({\hat{x}}_{g}\right)}$$ (12)

$$\text{SSIM}\left(g\right)=\frac{\left(2\mu_{g}^{\prime }{\hat{\mu }}_{g}^{\prime }+C_{1}\right)\left(2\mathrm{Cov}\left(x_{g}^{\prime },{\hat{x}}_{g}^{\prime }\right)+C_{2}\right)}{\left({\left(\mu_{g}^{\prime }\right)}^{2}+{\left({\hat{\mu }}_{g}^{\prime }\right)}^{2}+C_{1}\right)\left({\left(\sigma_{x_{g}}^{\prime }\right)}^{2}+{\left(\sigma_{{\hat{x}}_{g}}^{\prime }\right)}^{2}+C_{2}\right)}$$ (13)

where *C*_1_ = 0.01 and *C*_2_ = 0.03.

This normalization ensures that SSIM captures cell-wise distributional structure rather than absolute expression scale enabling meaningful comparison across genes and platforms whose measurement ranges differ by orders of magnitude. We computed SSIM on per-cell expression vectors rather than rasterized spatial images, and, hence, SSIM reflects cell-wise distributional similarity and does not depend on a 2-dimensional grid.

#### Root mean squared error

The RMSE [26] measures the error between measured and predicted gene expression. To compare prediction errors across genes with different expression scales and across technologies with different measurement ranges, RMSE is computed after *z*-score normalization of each gene across cells as follows:

$$z_{g,i}=\frac{x_{g,i}-\mu_{g}}{\sigma_{x_{g}}},\;{\hat{z}}_{g,i}=\frac{{\hat{x}}_{g,i}-{\hat{\mu }}_{g}}{\sigma_{{\hat{x}}_{g}}}$$ (14)

$$\text{RMSE}\left(g\right)=\sqrt{\frac{1}{N}\sum_{i=1}^{N}{\left(z_{g,i}-{\hat{z}}_{g,i}\right)}^{2}}$$ (15)

This normalization ensures that fair comparison of errors across genes and technologies by measuring how well cell-to-cell variation is recovered rather than absolute expression magnitude.

#### Wasserstein distance

Wasserstein distance [27] measures the discrepancy between the measured and predicted expression distributions across cells for each gene by measuring the shifts in distribution mass between the one-dimensional distributions. Wasserstein distance is computed after *z*-score normalization of each gene across cells as follows:

$$W\left(g\right)=\int_{-\infty }^{\infty }|F_{z_{g,i}}\left(t\right)-F_{{\hat{z}}_{g,i}}\left(t\right)|\mathrm{dt}$$ (16)

where $F_{z_{g,i}}\left(t\right)$ and $F_{{\hat{z}}_{g,i}}\left(t\right)$ denotes the cumulative distribution functions of $z_{g,i}$ and $\;{\hat{z}}_{g,i}$ respectively.

#### JS divergence

JS divergence [26] measures the similarity between the measured and predicted expression distributions across cells for each gene after converting them to probability distributions. JS is computed as follows:

$$p_{g}=\frac{x_{g}}{\sum_{i=1}^{N}x_{g,i}},{\hat{p}}_{g}=\frac{{\hat{x}}_{g,i}}{\sum_{i=1}^{N}{\hat{x}}_{g,i}},m=\frac{p_{g}+{\hat{p}}_{g}\;}{2}$$ (17)

where *p*_g_ and ${\hat{p}}_{g}\;$ denote the probability vector of spatial distribution using measured and imputed gene expression respectively.

$$\mathrm{KL}\;\left(a_{i}∥b_{i}\right)=\sum_{j=0}^{N}a_{\mathrm{ij}}\times \log\frac{a_{\mathrm{ij}}}{b_{\mathrm{ij}}}$$ (18)

where $a_{\mathrm{ij}}$ and $b_{\mathrm{ij}}$ denote the probabilities of gene i in cell j.

$$\mathrm{JS}=\frac{1}{2}\mathrm{KL}\;\left({\hat{p}}_{g}∥m\right)+\frac{1}{2}\mathrm{KL}\;\left(p_{g}∥m\right)$$ (19)

The average of PCC, RMSE, SSIM, Wasserstein distance, and JS across held-out genes is reported for performance evaluation. Higher PCC and SSIM and lower RMSE, Wasserstein distance, and JS indicate better prediction accuracy. We report PCC, SSIM, RMSE, and JS computed using the evaluation protocol provided in [26] for all methods.

#### Moran’s *I*

Moran’s *I* [30] is used to measure the spatial autocorrelation of gene expression across cells that is whether nearby cells tend to have similar gene expression. It is used to assess whether the imputed data preserve spatial structure present in the measured data.

#### SPARK-X

SPARK-X [28] is used to identify SVGs from the measured and imputed gene expression using spatial coordinates. Genes with FDR-adjusted *P* < 0.05 were considered significant SVGs. For benchmarking, we used the significant SVGs identified by SPARK-X from the raw measured spatial data as the reference set and quantified the recovery from each method’s imputed data using AUPRC, a threshold-free metric suitable for the imbalanced setting since SVGs represent only a subset of genes. Higher AUPRC indicates better recovery of the measured spatially variable signal. Dataset pairs for which no significant SVGs were detected in the measured spatial data were excluded because no reference SVGs could be defined.

#### Adjusted rand index

ARI is used for evaluation of spatial clustering using raw and imputed data. Considering that $\hat{Y}={\left\{\hat{y_{i}}\right\}}_{i=1}^{n}$ represents the identified spatial cell types and $Y={\left\{y_{i}\right\}}_{i=1}^{n}$ represents the ground-truth spatial domains from n cells, then ARI is calculated as:

$$\begin{array}{c} \mathrm{ARI}=\frac{\sum_{\mathrm{ls}}\left(\begin{array}{c} n_{\mathrm{ls}}\\ 2 \end{array}\right)-\frac{\sum_{l}\left(\begin{array}{c} n_{l}\\ 2 \end{array}\right)\sum_{s}\left(\begin{array}{c} n_{s}\\ 2 \end{array}\right)}{\left(\begin{array}{c} n\\ 2 \end{array}\right)}}{\frac{\sum_{l}\left(\begin{array}{c} n_{l}\\ 2 \end{array}\right)+\sum_{s}\left(\begin{array}{c} n_{s}\\ 2 \end{array}\right)}{2}-\frac{\sum_{l}\left(\begin{array}{c} n_{l}\\ 2 \end{array}\right)\sum_{s}\left(\begin{array}{c} n_{s}\\ 2 \end{array}\right)}{\left(\begin{array}{c} n\\ 2 \end{array}\right)}} \end{array}$$ (20)

where l and s denote the k cell types, $n_{l}=\sum_{i}^{n}I\left(\hat{y_{i}}=l\right)$, $n_{s}=\sum_{i}^{n}I\left(y_{i}=s\right)$, $n_{\mathrm{ls}}=\sum_{i,j}^{n}I\left(\hat{y_{i}}=l\right)I\left(y_{i}=s\right)$, and $I\left(x=y\right)=1$ when x=y, else $I\left(x=y\right)=0$.

#### Normalized mutual information

NMI is used for evaluation of spatial clustering agreement between raw and imputed data. Considering that $\hat{Y}={\left\{\hat{y_{i}}\right\}}_{i=1}^{n}$ represents the identified spatial cell types and $Y={\left\{y_{i}\right\}}_{i=1}^{n}$ represents the ground-truth spatial domains from n cells, then NMI is calculated as:

$$\mathrm{NMI}=\frac{2\times \mathrm{MI}\left(Y,\hat{Y}\right)}{H\left(Y\right)+H\left(\hat{Y}\right)}$$ (21)

where $\mathrm{MI}\left(Y,\hat{Y}\right)$ denotes the mutual information between Y and $\hat{Y}$ and $H\left(Y\right)$ and $H\left(\hat{Y}\right)$ denotes their entropies.

#### Silhouette coefficient

Silhouette coefficient measures clustering performance using pair-wise distance within and between identified clusters, and a higher value of the score denotes well-defined clusters. It is calculated as:

$$\begin{array}{c} \mathrm{Silhouette}\;\mathrm{Coefficient}=\frac{1}{k}\sum_{l=1}^{k}\frac{1}{n_{l}}\left(\sum_{x\;\epsilon \;C_{l}}\frac{b\left(x\right)-a\left(x\right)}{\max\left(b\left(x\right),a\left(x\right)\right)}\right) \end{array}$$ (22)

where k is the number of clusters, $n_{l}$ denotes number of samples in cluster l, $a\left(x\right)$ denotes average distance between sample x and all samples from same cluster, $b\left(x\right)$ denotes average distance between sample x and all samples from nearest cluster, and $C_{l}$ denotes cluster l.

### Computational cost

We evaluated the computational cost for each method on 2 datasets with different scales: osmFISH_Zeisel dataset pair and NanoString_GSE dataset pair. On osmFISH_Zeisel dataset pair, SpaGene required 5 min 28 s with 0.456 GB peak graphics processing unit (GPU) memory, compared with SpaGE (18 s, GPU not utilized), gimVI (7 min 44 s, 0.349 GB), Tangram (52 s, 0.544 GB), spRefine (9 min 1 s, 0.356 GB), VISTA (18 min 29 s, 0.989 GB), and stDiff (22 min 4 s, 1.03 GB). On NanoString_GSE dataset pair, SpaGene required 49 m 18 s with 1.06-GB peak GPU memory, compared with SpaGE (17 min 31 s, GPU not utilized), gimVI (1 h 24 min, 1.394 GB), Tangram (~24 h on central processing unit after GPU out-of-memory), spRefine (24 min, 0.866 GB), VISTA (5 h 20 min, 1.331 GB), and stDiff (8 h, 1.823 GB). The experiments were conducted on a workstation running Ubuntu 22.04.5 LTS with an NVIDIA A40 GPU, AMD EPYC 74F3 24-core central processing unit and 64-GB RAM. These results indicate that SpaGene introduces moderate computational overhead relative to lightweight methods but remains more practical than deep-learning baselines on large datasets (Table S11).

## Data Availability

All datasets used for evaluation are publicly available. NanoString CosMX SMI dataset can be downloaded from https://nanostring.com/products/cosmx-spatial-molecular-imager/ffpe-dataset/. GSE dataset can be downloaded from https://www.ncbi.nlm.nih.gov/geo/query/acc.cgi?acc=GSE131907. MERFISH, Moffitt, osmFISH, AllenSSp, AllenVISp, Zeisel, seqFISH, and STARmap datasets can be downloaded from the public repository https://zenodo.org/records/3967291. Xenium_breast dataset can be downloaded from https://www.ncbi.nlm.nih.gov/geo/query/acc.cgi?acc=GSM7780153, and GSE_breast dataset can be downloaded from https://www.ncbi.nlm.nih.gov/geo/query/acc.cgi?acc=GSE243280. The SpaGene method is provided as an open-source Python package on GitHub: https://github.com/asbudhkar/SpaGene. A public website is also provided for user-friendly SpaGene overview: https://asbudhkar.github.io/SpaGene-project-website/.
