# Supplementary material for: SpaGene: A Deep Adversarial Framework for Spatial Gene Imputation
Source: Comput Struct Biotechnol J. 2026 May 15;35(1):0102. doi: 10.34133/csbj.0102 (PMC13176606; doi:10.34133/csbj.0102)

Supplementary Figure 1

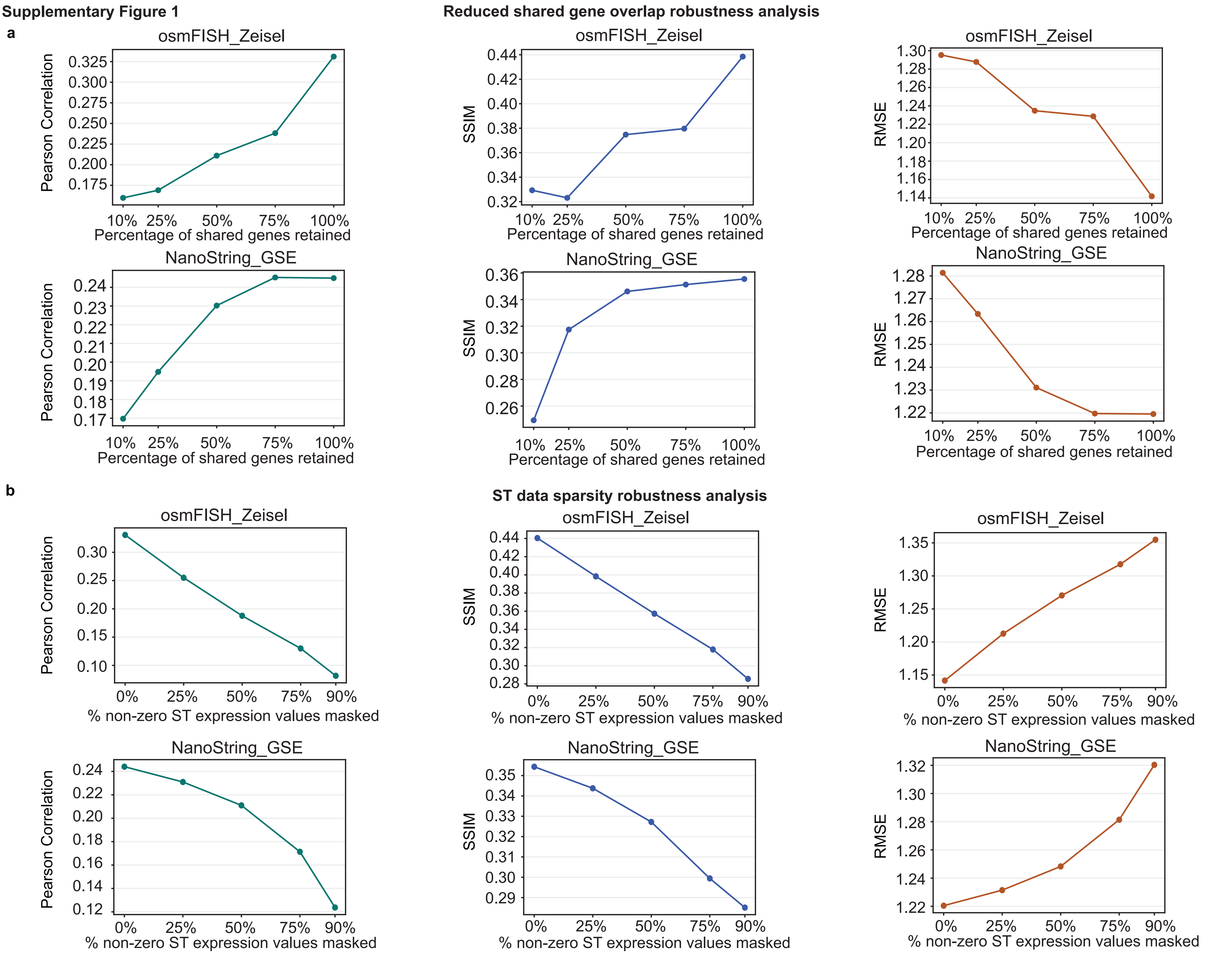

Supplement: Supplementary 1 — Figs. S1 to S5 Tables S1 to S11 [file csbj.0102.f1.zip › Supplementary Fig1.pdf]
