# Supplementary material for: SpaGene: A Deep Adversarial Framework for Spatial Gene Imputation
Source: Comput Struct Biotechnol J. 2026 May 15;35(1):0102. doi: 10.34133/csbj.0102 (PMC13176606; doi:10.34133/csbj.0102)
Supplement: Supplementary 1 — Figs. S1 to S5 Tables S1 to S11 [file csbj.0102.f1.zip › Supplementary Legends.docx]

**Supplementary Figure 1:** Robustness analyses on the osmFISH_Zeisel and NanoString_GSE dataset pairs. (a) Reduced shared gene overlap analysis showing performance of SpaGene when only 10%, 25%, 50%, 75%, or 100% of shared genes were retained. (b) ST data sparsity analysis showing SpaGene performance when 0%, 25%, 50%, 75%, or 90% of the originally non-zero ST expression values were masked. Average PCC, SSIM, and RMSE are shown.

**Supplementary Figure 2:** Spatial autocorrelation shift analysis for held-out genes in the MERFISH_Moffitt dataset pair. (a) Histogram of ΔMoran’s I values between SpaGene imputed and measured expression across held-out genes. (b) Cumulative distribution of |ΔMoran’s I|.

**Supplementary Figure 3:** Downstream clustering and spatially variable gene analysis. (a) UMAP visualization for clustering using raw measured genes, imputed measured genes (only measured genes in raw data), and imputed all genes (both measured genes in raw data and newly imputed genes). ARI, NMI, and silhouette score values are reported for overall clustering comparison. Top row shows all cells and bottom row shows non-tumor cells. (b) SVG comparison between raw genes and imputed measured genes (only measured in raw data). Panels show significant SVG counts identified by SPARK-X with Jaccard overlap, overlap of top-k SVGs, and gene-wise Moran’s I agreement between raw and imputed data.

**Supplementary Figure 4:** Latent neighborhood recall (k=15, m=45) across translation (STARmap AllenVISp dataset pair). (a) ST versus ST $\to$ SC neighborhood recall. (b) ST versus ST $\to$ SC $\to$ ST neighborhood recall. (c) SC versus SC $\to$ ST neighborhood recall. (d) SC versus SC $\to$ ST $\to$ SC neighborhood recall. Lines denote per-fold mean recalls for cross-validation.

**Supplementary Figure 5:** Comparison of frozen two-stage training and end-to-end optimization on osmFISH_Zeisel and NanoString_GSE dataset pairs. Validation PCC trajectories across training epochs are shown for frozen and end-to-end training as mean ± standard deviation across three random seeds.

**Supplementary Table:** Sheet1: PCC, SSIM, RMSE, Wasserstein distance, and JS results of each of the methods across 8 datasets. Sheet2: Significance results: FDR corrected p-value using paired t-test and Wilcoxon signed-rank test across 8 datasets. Sheet3: SPARK-X SVG recovery AUPRC for each method. Sheet4: Comparison of frozen two-stage training and end-to-end optimization on osmFISH_Zeisel and NanoString_GSE dataset pairs. Sheet5: Summary of datasets used in this study. Sheet6: Hyperparameter sensitivity results on MERFISH_Moffitt dataset pair. Sheet7: Hyperparameter sensitivity results on NanoString_GSE dataset pair. Sheet8: Loss sensitivity results on MERFISH_Moffitt dataset pair. Sheet9: Loss sensitivity results on NanoString_GSE dataset pair. Sheet10: Baseline method source repository and version. Sheet11: Computational cost comparison on osmFISH_Zeisel and NanoString_GSE dataset pairs.
