# Supplementary figures and images for: SpaGene: A Deep Adversarial Framework for Spatial Gene Imputation
Source: Comput Struct Biotechnol J. 2026 May 15;35(1):0102. doi: 10.34133/csbj.0102 (PMC13176606; doi:10.34133/csbj.0102)

**Supplementary Figure 3**

**a.**

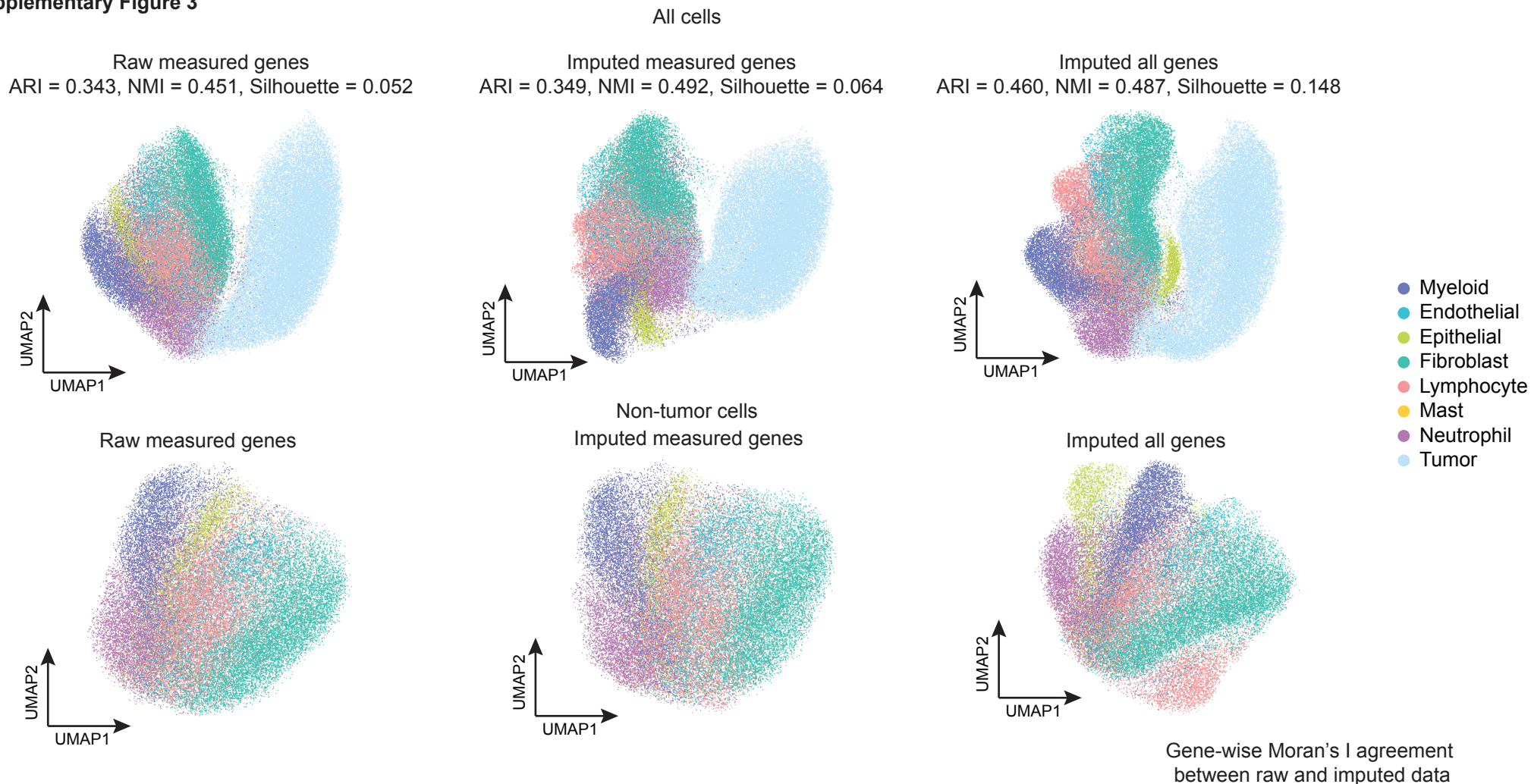

**b.**

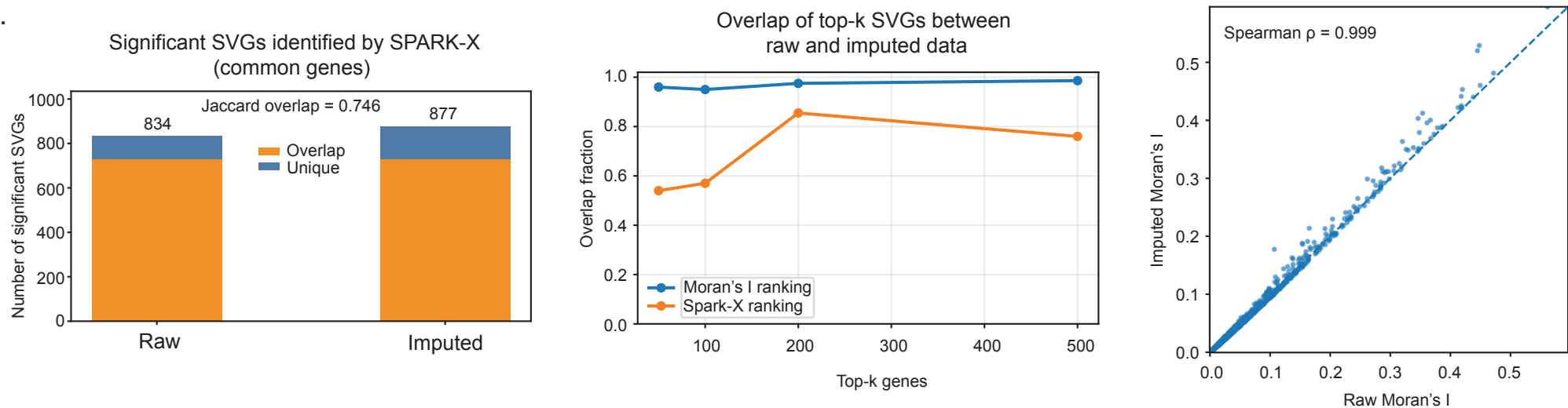

Supplement: Supplementary 1 — Figs. S1 to S5 Tables S1 to S11 [file csbj.0102.f1.zip › Supplementary Fig3.pdf]

Supplementary Figure 4

STARmap AllenVISp dataset pair neighborhood recall (k=15, m=45)

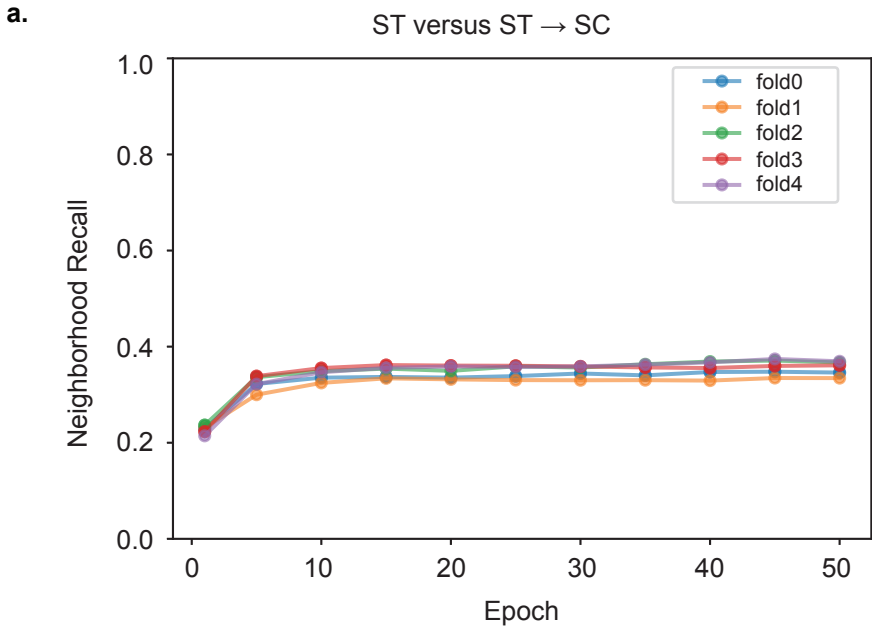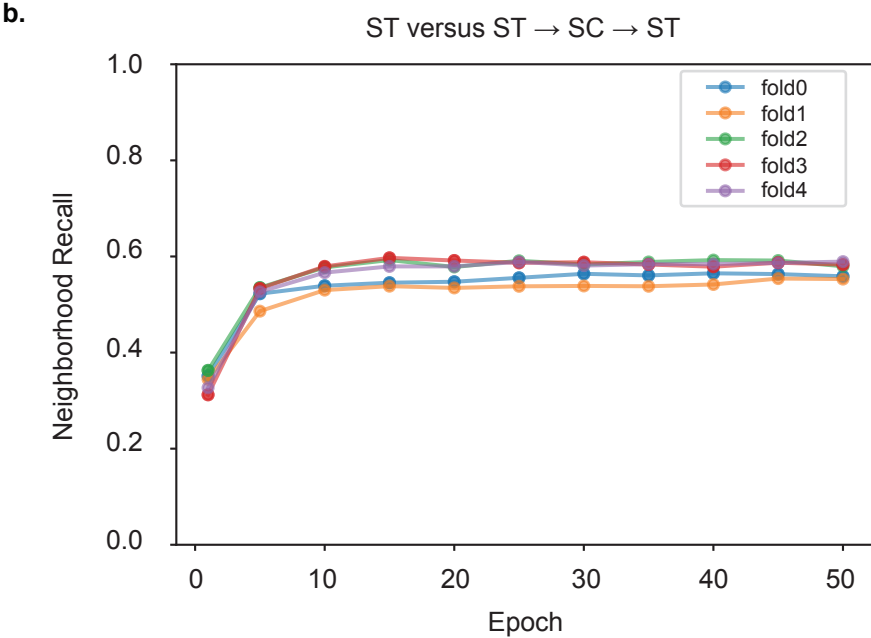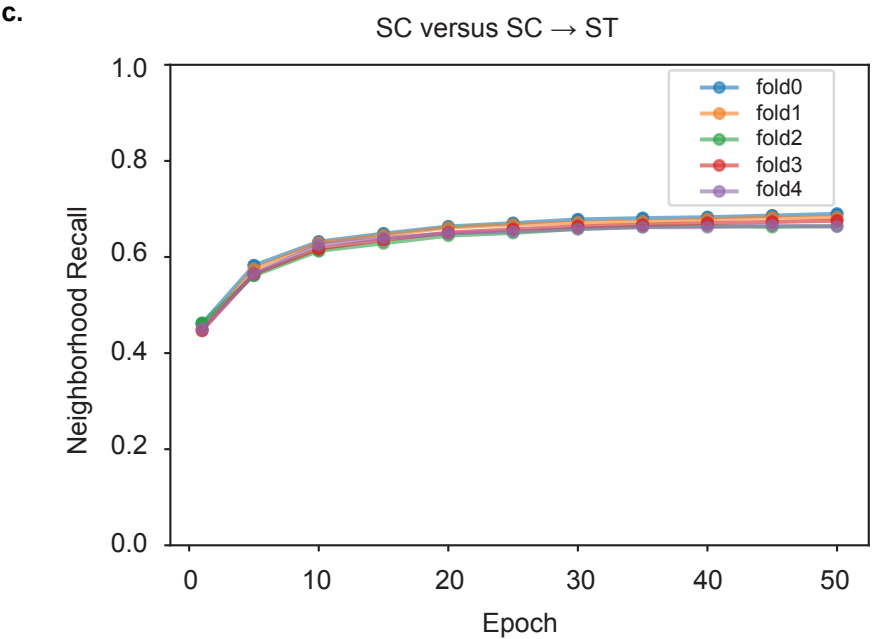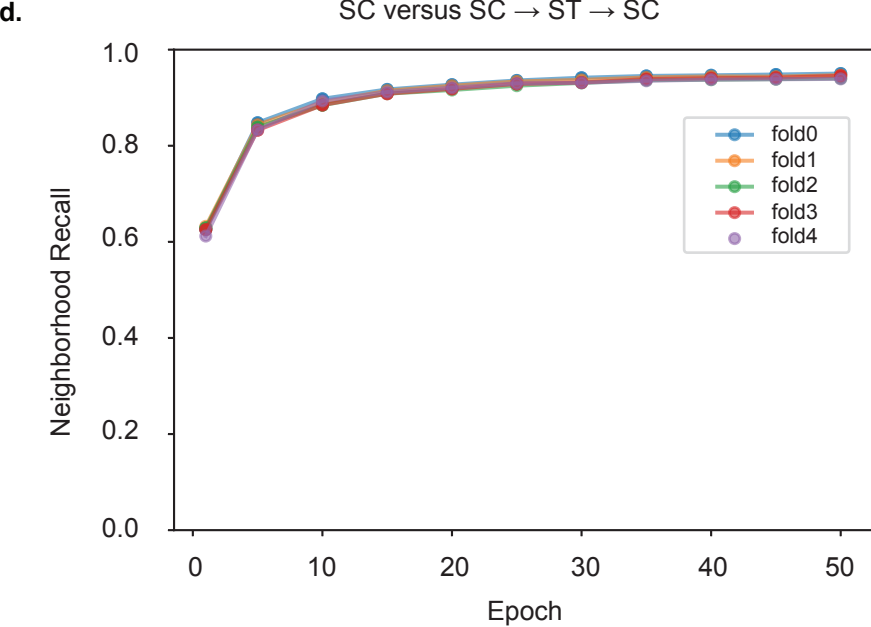

Supplement: Supplementary 1 — Figs. S1 to S5 Tables S1 to S11 [file csbj.0102.f1.zip › Supplementary Fig4.pdf]

**Supplementary Figure 5**

Frozen versus end-to-end training

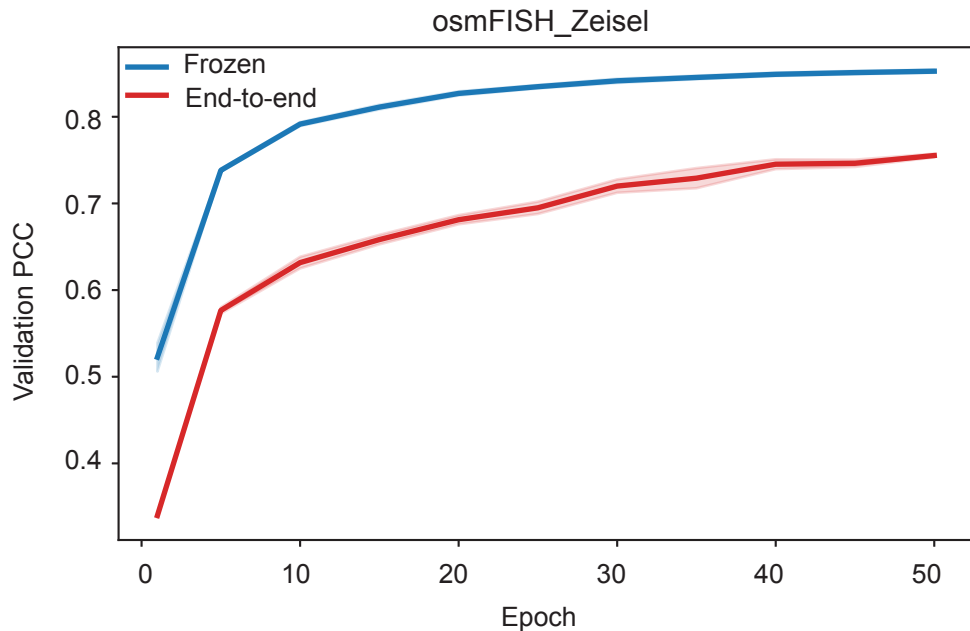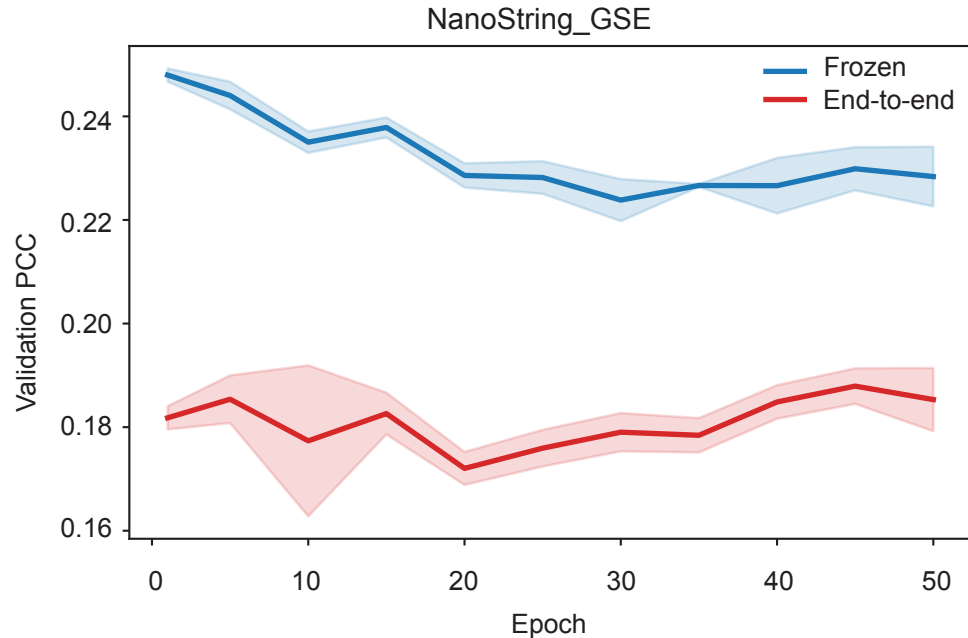

Supplement: Supplementary 1 — Figs. S1 to S5 Tables S1 to S11 [file csbj.0102.f1.zip › Supplementary Fig5.pdf]
